# The Demon, Alcohol

**Published:** 1897-11

**Authors:** F. E. Daniel

**Affiliations:** Austin, Texas


### TEXAS MEDICAL JOURNAL.

ESTABLISHED JULY, 1885.

PUBLISHED MONTHLY.—SUBSCRIPTION $2.00 A YEAR.

VOL. XIII. AUSTIN, NOVEMBER, 1897. No. 5.

### Original Contributions.

For the Texas Medical Journal.

### THE DEMON, ALCOHOL.

AN ADDRESS OF WELCOME ON BEHALF OF THE MEDICAL PROFESSION, DELIVERED BEFORE THE WOMAN'S CHRISTIAN TEMPERANCE UNION OF TEXAS.

BY F. E. DANIEL, M. D., AUSTIN, TEXAS.

*Women of the Christian Temperance Union:*

Upon me devolves the pleasing duty of extending to you, on behalf, and in the name of, the medical profession of Austin, a welcome. We welcome you to our hearts and homes in the fair Capital City, the seat of learning, the home of wealth, refinement and Christian charity; a city where intemperance is but a name, and drunkenness is unknown.

Assured, as you are, of the sympathy of the better element of the medical profession in the grand work for humanity you are doing, I am here to pledge you their aid, support and cooperation, and to bid you God speed. The medical profession are fast becoming your allies in the great Temperance Reform movement. Satisfied, as many of them are, from long experience and observation, that alcohol is not an absolutely indispensable requisite in therapeutics, they are prescribing it less and less each year. That it has its uses, and employed with judgment and discretion, it is a valuable resource in medicine, cannot be denied; but under the erroneous impression that it is a food and stimulant, it has been greatly abused, and I have seen it administered in such excess that the "remedy" has overshadowed the disease, and the patient, instead of dying of pneumonia, for instance, has died of acute alcoholic poisoning. Every well read physician knows that only about two ounces of absolute alcohol can be consumed, burned, in the human stomach, appropriated to the uses of the economy in twenty-four hours; and any amount given in excess of this enters the blood unchanged, and becomes a poison, the elimination of which devolves upon the heart, liver, lungs and kidneys, frequently overwhelmning them and causing death. I have said that alcohol is not a food nor a stimulant. This will be a surprise to many, for it has stood at the head of the list of stimulants from time immemorial. It is not a food, but by lessening the tissue waste, ("metabolism" technically), it lessens the demand for food. After a brief exaltation or stimulation, whereby the physician is enabled, often, to bridge certain crises, alcohol becomes a powerful depressant of vital power; and it is the experience of the best observers, that those who use it habitually, even in moderation, can do less work, sustain less fatigue, and withstand exposure less, than those who do not use it, and the power of resistance to disease is less; when attacked with disease the former yield, when the latter would recover.

A gratifying sign of the times, and one which should give us great encouragement, is the marked decline in the consumption of alcohol throughout the world. It has been greater in America than elsewhere, and, since 1888, amounts to about 30 per cent., nearly one-third. That is, as compared with 1888, the total consumption of alcohol in the United States in 1896, is one gallon per capita less. Think, a moment, what this means. There are 70,000,000 population in the United States. That indicates that 70,000,000 gallons of alcohol less were consumed in 1896 than in 1888. Suppose that there is one man to every seven of the population. It means that each man drank in 1896 seven gallons less than formerly. It means that 70,000,000 gallons of alcohol (absolute, "proof spirit," from which liquors are made, diluted), at, say, $2 per gallon, represents a saving on this commodity of $140,000,000, a sum sufficient to build thirty granite palaces like the Capital at Austin; a sum sufficient to support every Christian minister in the world.

Soldiers of the Grand Army of Temperance, you are engaged in a crusade almost as sacred as that which, in the twelfth century, had for its object the rescue of the Holy Sepulchre from infidel hands. In those days the men, husbands, brothers, sons, lovers, fired by religious zeal, were sent forth by the women, consecrated to the cause by their prayers, tears and blessings. To-day the women, enlisted under the banner of Temperance, and in the name of humanity, go forth to battle, and the men look on and applaud. You have a foe to meet, more terrible, more remorseless than the Saracen; a despot, who annually demands and destroys the souls and bodies of thousands of our brightest and best. You are invading the domains of the demon Drink. You are to assault the stronghold of the Devil in his favorite role, the whisky traffic; a stronghold entrenched behind millions of capital, and fortified by political power. The struggle will be long and bitter; but as there is a just God, who controls the destinies of man, whom He created for a nobler fate, the victory will be yours in the end. Do not despair.

We read in Grecian mythology, that in prehistoric times there was, in the Isle of Crete, a monster of most hideous mein, half man and half beast,—a fitting emblem of drunkenness, the fabled Minotaur,—who terrorized the inhabitants of that country. He devoured human beings, and demanded tribute in flesh and blood, and annually Athens paid the terrible tax, by a levy on the youths and maids of Greece. God raised up a deliverer, however, and the monster was slain by the young Greek, Theseus. But, *to a woman* belongs the victory; it was a woman, the beautiful Princess Ariadne, who put into his hands the sword with which the monster was slain. She gave him, too, a thread, by following which he might escape from the Labarynth, the home of the monster, one from which no living human being had ever escaped. For his valor the princess rewarded Theseus with her hand in marriage. You, women of the Christian Temperance Union, have just such a monster to encounter and overthrow—the modern Minotaur, the demon Drink. He has his gilded labarynths, the infernal saloons, in every village, town, city and hamlet in the civilized world; in some towns, three or four to every street. Woe to the unwary who enter there, for there is no thread that will ever lead him to freedom when once entrapped in these gilded hells. They sing the siren song to our youth. They are made seductive by bright lights, pretty pictures and the strains of sweet music. It may well be inscribed over the portals:

"Who enters here, leaves hope behind."

And yet, legislation is powerless to mitigate the evil. Prohibition does not prohibit, taxation does not lessen the sales. The falling off in the annual consumption of distilled spirits alluded to above cannot fairly be attributed to any restrictive legislation. (It must be remembered that all manufactured spirit is not consumed by drink, but a large part is used in the arts and sciences.) It is human nature to want that which is hard to get, and it is man's nature, some men, at least, to have whiskey at whatever cost, regardless of the privation and suffering the indulgence of their accursed appetite entails on their innocent dependent ones. The higher the price of whiskey, the more money is spent for it. The press attribute the decline to hard times. It is a mistake. Why not give the advance in civilization, the temperance reform movement, the Women's Christian Temperance Union, and doctors, credit for part of it, at least? Public sentiment has had a great deal to do with the lessening of drunkenness. Twenty years ago the habit of treating was almost universal. It was a courtesy, which, when extended, few dared refuse. It required a brave man to decline to drink when asked. It gave offense, and not infrequently led to strife. It was not uncommon to see our best citizens enter saloons, and to see them more or less under the influence of liquor. Now, no self-respecting man will frequent a saloon. No business man will employ a drunkard. I believe that the saloon is still the great evil of the day, and is responsible for more misery, sin and suffering than all other causes combined. But, how deal with it? There is but one way,—make it unpopular. Make it odious. Let drunkenness and dishonor be synonymous. Let the odor of liquor on the breath be a badge of disgrace. Let every young woman pledge herself that she will not receive the attentions of no man on whose breath is the taint of the accursed, soul-destroying poison. In this way the youth, at least, can be saved from the dangers of drink.

May some fair Ariadne be raised up among you to place in your hands the consecrated sword of Theseus, with which, at one stroke, the demon drink may be destroyed, and man forever ransomed from the thraldom of his power. May God give you grace and strength to *strike*, till the shadow that now enshrouds so many homes may be lifted. May He hold up your hands, "even to the going down of the sun," and the battle is won,—for Christ and humanity.

